# Identifying distinct profiles of impulsivity for the four facets of psychopathy

**DOI:** 10.1371/journal.pone.0283866

**Published:** 2023-04-14

**Authors:** Samuel J. West, Elena Psederska, Kiril Bozgunov, Dimitar Nedelchev, Georgi Vasilev, Nicholas D. Thomson, Jasmin Vassileva

**Affiliations:** 1 Department of Psychology, Virginia State University, Petersburg, VA, United States of America; 2 Department of Surgery, Virginia Commonwealth University, Richmond, VA, United States of America; 3 Department of Cognitive Science and Psychology, New Bulgarian University, Sofia, Bulgaria; 4 Bulgarian Addictions Institute, Sofia, Bulgaria; 5 Department of Psychiatry, Virginia Commonwealth University, Richmond, VA, United States of America; Medical University of Vienna, AUSTRIA

## Abstract

Psychopathy comprises antagonistic personality traits and antisocial behaviors that are associated with critical outcomes for the individual and society (e.g., violent behavior). Since its inception, impulsivity has been theorized as a core feature of psychopathy. Research supports this assertion, yet psychopathy and impulsivity are both multifaceted constructs. As such, the associations commonly observed between psychopathy and impulsivity may obscure more nuanced profiles of impulsivity that are only observable at the facet-level. To address this gap in the literature, we collected data from a community sample using a clinical psychopathy interview along with dispositional and neurobehavioral measures of impulsivity. We regressed each of the four facets of psychopathy onto eight impulsivity variables. We followed these analyses with bootstrapped dominance analyses in order to determine which of the impulsivity variables shared the most variance with each psychopathy facet. Our analyses revealed that positive urgency was the most important aspect of impulsivity to all four facets of psychopathy. We further identified distinct profiles of impulsivity linked to each psychopathy facet–the interpersonal facet was typified by sensation seeking and temporal impulsivity. The affective and lifestyle facets were both typified by general trait impulsivity and affective impulsivity. The antisocial facet was typified by affective impulsivity and sensation seeking. Such distinct profiles of impulsivity suggest that specific behaviors linked with each facet (e.g., manipulativeness and the interpersonal facet) may be explained in part by the distinct forms of impulsivity aligned with them.

## Introduction

Psychopathy is a collection of dispositional features reflecting impairments in affective and interpersonal domains, as well as impulsive, irresponsible and antisocial behavioral patterns associated with increased susceptibility to both risky and violent behaviors [1]. In addition to its strong connection to risky activities, psychopathy is a reliable predictor of various externalizing behaviors such as substance use disorders [2, 3]. Given that psychopathy has been consistently connected to poor treatment outcomes [4–6], understanding its key features and their correlates can inform the development of novel and effective treatment alternatives that can address the main limitations of traditional treatment interventions. Psychopathy has been conceptualized through various theoretical models; one such model is the four-facet model of psychopathy.

### Four facet model of psychopathy

Psychopathy is commonly operationalized by the Psychopathy Checklist inventories including the Psychopathy Checklist–Revised (PCL-R; [7]), the Psychopathy Checklist: Youth Version (PCL:YV; [8]) or the Psychopathy Checklist: Screening Version (PCL:SV; [9]), considered the “gold standard” for measuring psychopathy. Early versions of the PCL measures conceptualized psychopathy as comprised of two factors [10]. Factor 1 captures the core interpersonal and affective features of psychopathy, whereas Factor 2 is related to the impulsive and antisocial domains [11]. However, other psychometric studies indicate that four lower-order facets might better capture the latent structure of the psychopathy construct, reflecting the nuanced variability among psychopathic traits [1]. The four-facet model of psychopathy has been well validated and replicated in all PCL inventories [1, 12, 13]. Recent studies utilizing the four-facet model have emphasized that addressing the separate psychopathy dimensions might be beneficial in expanding our knowledge regarding the complex and often differential relationships between psychopathy facets and other criterion variables (e.g., impulsivity) central to the study of psychopathy [14–17]. This approach is likely to be even more important in the case of common correlates of psychopathy that are also multifaceted constructs, such as impulsivity, given that the associations among higher-order traits necessarily obscure distinct patterns of covariance that may be observed at the facet level [18].

### Forms of impulsivity

Impulsivity is generally defined as a stable behavioral pattern associated with rapid and unplanned reactions to various external and internal stimuli [19]. Two overarching domains of impulsivity are reflected in the literature: trait impulsivity and neurobehavioral impulsivity. Trait impulsivity may refer to measures of *general impulsivity* such as that measured by the Barratt Impulsiveness Scale-11 (BIS-11; [20]) or specific facets of trait impulsivity linked to positive or negative emotional states (*affective urgency)* such as those measured by the UPPS Impulsive Behavior Scale (UPPS; [21]). Such measures capture stable individual dispositions towards impulsive behavior in various contexts.

In contrast to trait impulsivity, neurobehavioral components of impulsivity are more state-dependent, susceptible to environmental influences, and are usually measured by performance-based laboratory tasks [3, 22]. Neurobehavioral impulsivity can be further divided in specific subcomponents. *Decisional impulsivity* reflects reward-driven, disadvantageous and risky decision-making [23]. Common tasks for capturing this domain incorporate delay discounting tasks such as the Monetary Choice Questionnaire (MCQ; [24]), computerized gambling tasks such as the Iowa Gambling Task (IGT; [25]) and the Cambridge Gambling Task (CGT; [26]), which evaluate reward-driven decision-making under risk (i.e., CGT) and ambiguity (i.e., IGT). *Impulsive action* is the second component of neurobehavioral impulsivity reflecting impairments in rapid response inhibition [27]. Common tasks for measuring impulsive action include paradigms similar to the Go/No-Go task [28, 29]. Measures of trait and neurobehavioural impulsivity typically do not correlate, suggesting they represent distinct dimensions of the impulsivity construct [30, 31]. However, this list of measures is far from exhaustive, as many different measures have been used in both domains for these and other forms of impulsivity. Regardless of the domain, substantial literature has evinced robust links between psychopathy and most forms of impulsivity.

### Psychopathy and impulsivity

Impulsivity has long been considered a central feature of psychopathy [7, 32]. Studies reveal that both trait [7, 33–36] and neurobehavioral domains [37–42] of impulsivity are related to psychopathy. However, these studies largely employ different definitions and measures of impulsivity and psychopathy and have produced some conflicting findings. Some work indicates that trait impulsivity is more strongly related to the behavioral dimensions of psychopathy and weakly or inversely associated with the affective and interpersonal characteristics [7, 33, 35, 36, 43, 44]. This suggests that dispositional impulsivity might be considered a key aspect of the lifestyle and antisocial domains of psychopathy [34, 43], but plays insignificant role for the affective and interpersonal deficits associated with psychopathy. Research examining the links between the affective urgency components of impulsivity and psychopathy has produced more nuanced evidence, as negative urgency is related to Factor 1 of psychopathy (comprising the lifestyle and antisocial facets), but inversely related (or not related) to Factor 2 (comprising the affective and interpersonal facets) [35, 43, 45–49]. Studies testing the relationship between psychopathy and positive urgency are fewer, but reflect a similar pattern of associations primarily with Factor 1 traits of psychopathy [43, 46, 49]. However, some work has identified positive associations between the Factor 1 traits of psychopathy and positive urgency [46]. Similarly unclear findings exist regarding the connection between neurobehavioral impulsivity and psychopathy.

Many studies indicate that psychopathy is also associated with neurobehavioral dimensions of impulsivity. Indeed, individuals with greater psychopathy scores are characterized by disadvantageous decision-making which is reflected as increased risk-taking and impaired feedback learning [37, 38, 42, 50–53]. However, previous work has produced somewhat inconsistent findings with some studies suggesting impaired inhibition of motor reactions [39, 40, 54, 55], whereas others reported intact response inhibition in individuals diagnosed with psychopathy [39, 56–58]. Inconsistencies across findings may be explained by the multidimensional nature of psychopathy, as the majority of the studies investigating the relationships between psychopathy and neurobehavioral impulsivity examined these associations only at the domain-level (i.e., PCL sum scores). However, several studies have accounted for the distinct psychopathy dimensions and reported that only the lifestyle and antisocial domains are associated with both risky and disadvantageous decision-making [37, 59, 60] and impaired response inhibition [41, 61, 62]. In contrast, the interpersonal and affective dimensions of psychopathy were associated with higher inhibition capacity [41, 61, 62].

### Current study

A substantial body of literature indicates that impulsivity is a critical component of psychopathy. Despite this literature, there are conflicting findings regarding the relationship between the components of psychopathy and different forms of impulsivity. However, much of this work has also neglected to comparatively model different facets of impulsivity alongside the lower-order facets of psychopathy. Given that trait impulsivity and neurobehavioral impulsivity represent distinct components of impulsivity, one crucial step forward in understanding psychopathy is to examine the potential for the facets of psychopathy to reflect distinct profiles of impulsivity. In the current study we conducted a series of exploratory regressions and dominance analyses utilizing a broad array of trait and neurobehavioral impulsivity measures in order to provide some clarity regarding the nature of the associations among impulsivity and psychopathy at the facet level. Our approach allowed us to address this gap in the literature by examining the associations between these distinct forms of impulsivity and facets of psychopathy while controlling for the other forms of impulsivity in our models. All data and code used in our analyses are publicly available on the Open Science Framework (https://osf.io/ejdmk/files).

## Methods

### Participants

Participants were recruited from a larger study on impulsivity among substance users in Bulgaria via flyers placed at substance abuse clinics, therapeutic communities, social venues, as well as through the study’s web page and Facebook page. Participants were initially screened via telephone on their medical and substance use histories. The original sample comprised 793 participants, but only a subset of these, *N* = 479, had complete data on the variables of interest to this investigation. Of these participants, 64.70% were male and 55.53% were former substance users, whereas the remaining participants were non-users. Participants were 29.59 years of age on average (*SD* = 7.46). See S1 File for full details about the participants in our sample.

### Measures

#### Psychopathy facets

We measured psychopathy using the Psychopathy Checklist: Screening Version (PCL: SV; [7]). The PCL:SV is a semi-structured interview aimed at assessing 12 characteristics of psychopathy scored on a 3-point rating scale: 0 = absent, 1 = somewhat present, 2 = definitely present, [9]. A factor analysis of the Bulgarian version of the PCL:SV with a subset of the current Bulgarian sample revealed adequate fit to the data in a previous analysis [63]. The PCL:SV uses a four-facet structure of psychopathy, where the interpersonal (e.g., grandiosity, deceitfulness, superficial charm) and affective (e.g., lack of empathy, lack of remorse, failure to accept responsibility) facets comprise Factor 1 and the lifestyle (e.g., impulsivity, irresponsibility, lack of realistic long-term goals) and antisocial (e.g., poor behavioral controls, antisocial behavior in adolescence and adulthood) facets comprise Factor 2 (e.g., [64]). We computed scores for each of the four psychopathy facets as the mean of the three items comprising each facet.

#### General impulsivity

We assessed general trait impulsivity using the Barratt Impulsiveness Scale-11 (BIS-11; [20]). This measure asked participants to self-report on how often each of thirty scenarios (e.g., “I don’t ‘pay attention”) occurred to them on a scale of 1 (rarely/never) to 4 (almost always/always). We computed general impulsivity scores as the sum of all participant responses to the BIS-11. Psychopathy is positively linked with general impulsivity measured using the BIS-11 (e.g., [44]).

#### Affective impulsivity

We measured three distinct components of affective impulsivity: positive urgency, negative urgency, and lack of premeditation using the Urgency, (lack of) Premeditation, (lack of) Perseverance, Sensation Seeking, and Positive Urgency Impulsive Behavior Scale (UPPS-P; [65]). This 59-item measure asked participants to self-report the extent to which they agreed with each statement (e.g., “When I am really ecstatic I tend to get out of control”) on a scale of 1 (Disagree Strongly) to 4 (Agree Strongly). Participant scores were computed as the sum of all items that comprised the Lack of Premeditation (11 items), positive urgency (14 items), and negative urgency (12 items) subscales. These three facets of trait impulsivity are positively associated with psychopathy [48]. However, we did not include lack of perseverance in our analyses as this variable shows divergent associations across genders (e.g., is only associated with psychopathy in females) and our current sample did not have enough female respondents to make such comparisons [48]. In addition, we excluded the sensation seeking subscale from the analyses and instead utilized the complete Sensation Seeking Scale (SSS; [66]) which provided a more thorough assessment of this impulsivity facet.

#### Sensation seeking

We measured sensation seeking using the Sensation Seeking Scale (SSS; [66]), which is a 40-items forced choice self-report questionnaire. This measure asked participants to indicate their preference for one of two scenarios (e.g., 1 = “I like wild uninhibited parties”, 0 = “I prefer quiet parties with good conversation”). The sum of participant responses was used as their sensation seeking score. Elevated sensation seeking is one central aspect of psychopathy (e.g., [67]).

#### Decisional impulsivity

We measured decisional impulsivity as the quality of decision-making observed during the Cambridge Gambling Task (CGT; [26]). CGT is a computerized task assessing decision-making under risk, which does not require learning. Participants were presented with 10 boxes colored red or blue and are asked to guess whether a yellow token was hidden under a red or a blue box. The ratios of red:blue boxes varied from 1:9 to 9:1 in pseudorandom order. Participants earned points based on correct performance. In the second phase of the task participants were instructed to gamble points based on the confidence of their decisions, by selecting from an array of bets ranging from 5–95% of their earned points. Bets were presented in two conditions—ascending and descending order. For the purposes of the current study, we used the CGT Quality of Decision-Making index, which reflects betting on the more likely outcome of the two possible alternatives (i.e., red or blue boxes), as assessed by the percentage of instances in which the participant bet on the color that had the higher box ratio. Those with clinical levels of psychopathy demonstrate impaired levels of decision quality in relation to controls as measured by the CGT [68].

#### Response disinhibition / impulsive action

We measured response disinhibition as commission errors made during the Immediate Memory Task (IMT; [28]). The IMT is a continuous performance task in which series of five-digit numbers are shown on the computer screen for 500 ms each. Participants were instructed to respond only in cases when the presented stimulus is identical to the preceding one. Commission errors (i.e., false alarms), measuring incorrect responding to a non-target stimulus were used as an index of response disinhibition. Those diagnosed with anti-social personality disorder (ASPD), the clinical classification of psychopathy, exhibit poorer response inhibition as measured by the IMT (e.g., [69]).

#### Temporal impulsivity

We measured temporal impulsivity as delay discounting rates using the self-report Monetary Choice Questionnaire (MCQ; [24]). The questionnaire consists of 27 choices in which participants are asked to choose between a lesser-immediate amount of money and a greater-but-delayed amount available from 1 week to 6 months in the future. Participant discounting rates were estimated as the overall mean of *k*, calculated using the hyperbolic discounting function V = A/[1 + kD], where V is the value of reward A available at delay D. Participants with greater values of *k* exhibited greater temporal discounting (i.e., a preference for lesser-but-immediate rewards). We used the log-transformed values of the overall temporal discounting rate (i.e., MCQ Overall k) due to the non-normal distribution of MCQ scores in our sample. Greater temporal impulsivity measured as delay discounting is linked with greater levels of psychopathy [70].

### Procedure

The study was approved by the Institutional Review Boards of Virginia Commonwealth University and the Medical University of Sofia on behalf of the Bulgarian Addictions Institute. All participants gave written informed consent. All participants had to meet the following inclusion criteria: (1) age between 18 and 50 years; (2) Raven’s Progressive Matrices [71] estimated IQ higher than 75; (3) minimum of 8th grade education; (4) being able to read and write in Bulgarian; (5) HIV-seronegative status, determined by rapid HIV testing; (6) negative breathalyzer test for alcohol (Alcoscan AL7000) and negative urine toxicology screen for amphetamines, methamphetamines, cocaine, opiates, methadone, cannabis, benzodiazepines, barbiturates, and MDMA. Exclusion criteria included history of neurological illness, head injury with loss of consciousness of more than 30min, history of psychotic disorders, current treatment with opioid agonists and/or use of medications that affect impulsivity (i.e., neuroleptics, antidepressants, benzodiazepines). Testing was conducted by an experienced team of trained psychologists at the Bulgarian Addictions Institute in Sofia, Bulgaria. Data were collected in two sessions of approximately 4 hours each, conducted on two separate days. The assessment battery included a combination of clinical interviews, self-report questionnaires and computer-based neurobehavioral tests. The first session included assessment of substance use disorders, externalizing psychopathology (e.g., psychopathy, antisocial personality disorder, ADHD) and intelligence. The second session included completion of neurocognitive tasks and self-report measures of externalizing and internalizing personality traits and disorders (e.g., impulsivity, sensation seeking, depression, alexithymia). Participants were paid a total of 80 Bulgarian leva (approximately $50 USD) for participation in the study.

### Analytic approach

All analyses were conducted in R version 4.1.1. We examined the interrater reliability of our PCL variables using the irr package for R [72]. We utilized the *lm* function from base R to construct our initial multiple regression models. To examine the impulsivity profile of each facet of psychopathy we then subjected each regression to a dominance analysis. Dominance analysis is preferable to simple comparisons of coefficient weights and effect sizes because it tests every possible combination of predictors in a series of subset models that increase in complexity until all predictors are accounted for. The models tested in the current study contained eight predictor variables each and thus dominance was tested by examining every possible model starting from each predictor in a model on its own through each possible subset of predictor combinations (i.e., two through eight predictors).

In the current work we specifically tested predictors for complete dominance, which indicates that a given independent variable (e.g., negative urgency) shared more unique variance (i.e., R^2^) with the dependent variable than another (e.g., delay discounting) in every possible subset model at increasing levels of model complexity (i.e., the number of predictors included each step [73]). In the case that complete dominance could not be established we also examined general dominance, which simply compares the average contribution of all predictors across subset models. We also subjected our dominance analyses to 5,000 sample nonparametric bootstraps to examine the stability of our results. Given this approach, dominance analyses do not rely on traditional *p*-value statistics to determine dominance as some variables are likely to be significant in some subset models, but not others [74]. All dominance analyses were conducted using the dominanceAnalysis package for R [75]. Given that the data tested in our analyses was collected for another study protocol, this work comprises secondary data analysis [62].

## Results

### Descriptive statistics

All descriptive statistics are presented in Table 1. Zero-order bivariate correlations among all study variables are presented in S1 Table. Overall participant scores on the PCL:SV were 7.73 on average (*SD* = 6.48; range: 0–24). Forty-five of our participants met the PCL:SV cutoff designation (scores greater than 17) for psychopathy where an additional 71 met the more “relaxed” cutoff used by some researchers (scores greater than 12).” None of our variables demonstrated substantial skew or kurtosis (i.e., +/- 2.00) excepting the decision quality variable which was kurtotic. Some variables in this study exhibited univariate outliers (i.e., +/- 3 SD from the mean) which were Winsorized prior to our analyses.

**Table 1:** Descriptive statistics of all study variables.

Variable	Measure	*M*	*SD*	Min	Max	Skew	Kurtosis	Outliers	α
Affective	PCL:SV	0.59	0.61	0	2	0.80	-0.55	0	.76
Antisocial	PCL:SV	0.8	0.72	0	2	0.29	-1.37	0	.84
Lifestyle	PCL:SV	0.82	0.62	0	2	0.33	-1.03	0	.67
Interpersonal	PCL:SV	0.53	0.48	0	2	0.74	-0.16	7	.55
General Impulsivity	BIS	62.71	11.87	33	112	0.44	0.31	3	.86
Decision Quality	CGT	0.87	0.13	0	1	-1.67	3.72	12	-
Delay Discounting	MCQ	0.07	0.08	0	0.25	1.46	0.78	0	-
Commission Errors	IMT	38.85	14.07	3.31	79.7	-0.03	-0.62	0	-
Lack of Premeditation	UPPS-P	25.53	6.06	11	44	0.16	-0.20	1	.82
Negative Urgency	UPPS-P	25.29	7.43	10	46	0.40	-0.43	0	.92
Positive Urgency	UPPS-P	25.92	9.33	7	54	0.82	0.12	3	.89
Sensation Seeking	SSS	19.97	6.92	3	36	-0.18	-0.47	0	.84

*Notes*. BIS = Barratt Impulsiveness Scale, CGT = Cambridge Gambling Task, IMT = Immediate Memory Task, MCQ = Monetary Choice Questionnaire, PCL:SV = Psychopathy Checklist: Screening Version, SSS = Sensation Seeking Scale, UPPS-P = Urgency, Premeditation, Perseverance, Sensation Seeking, and Positive Urgency Impulsive Behavior Scale.

### Interrater reliability

To examine the interrater reliability among our PCL:SV data a second interviewer scored the PCL:SV for 154 participants from the full sample. We then estimated a two-way random effects single measure intraclass correlation coefficient (ICC) testing absolute agreement for each item as has been applied to PCL data in the past (e.g., [76]). All items demonstrated sufficient interrater reliability (i.e., all ICC values .71 or greater). See S2 Table for full interrater reliability statistics.

### Regression models

We estimated four multiple regression models with each of the four facets of psychopathy regressed onto the impulsivity variables. Although some of the impulsivity variables included in our models shared relatively strong covariances, our multicollinearity diagnostics suggested that this was not an issue for our analyses (i.e., all VIF values < 5; Tables 2–5). Model 1 (Table 2) implemented the interpersonal facet as the outcome variable. Model 1 revealed significant positive associations of delay discounting, positive urgency, and sensation seeking with the Interpersonal facet of psychopathy. Model 1 accounted for 16% of the variability in the interpersonal facet.

**Table 2:** Regression Model 1 results predicting the interpersonal facet.

Predictor	*β*	95% CI	*p*	*VIF*
Commission Errors	0.03	-0.06 – 0.11	.523	1.01
Decision Quality	-0.08	-0.17 – 0.01	.076	1.03
Delay Discounting	0.15	0.06 – 0.23	.001	1.03
General Impulsivity	0.04	-0.11 – 0.19	.603	2.97
Lack of Premeditation	0.00	-0.11 – 0.11	.996	1.69
Negative Urgency	-0.11	-0.25 – 0.04	.139	3.06
Positive Urgency	0.34	0.20 – 0.48	< .001	2.83
Sensation Seeking	0.16	0.07 – 0.26	.001	1.29

*Note*. VIF = Variance Inflation Factor.

**Table 3:** Regression Model 2 results predicting the affective facet.

Predictor	*β*	95% CI	*p*	*VIF*
Commission Errors	0.10	0.02 – 0.18	.014	1.01
Decision Quality	-0.10	-0.19 – -0.01	.024	1.03
Delay Discounting	0.12	0.03 – 0.20	.006	1.03
General Impulsivity	0.05	-0.10 – 0.20	.500	2.97
Lack of Premeditation	0.01	-0.10 – 0.12	.857	1.69
Negative Urgency	-0.05	-0.19 – 0.09	.508	3.06
Positive Urgency	0.38	0.24 – 0.52	< .001	2.83
Sensation Seeking	0.05	-0.05 – 0.14	.316	1.29

*Note*. VIF = Variance Inflation Factor.

**Table 4:** Regression Model 3 results predicting the lifestyle facet.

Predictor	*β*	95% CI	*p*	*VIF*
Commission Errors	0.08	0.00 – 0.16	.038	1.01
Decision Quality	-0.04	-0.12 – 0.04	.341	1.03
Delay Discounting	0.13	0.05 – 0.20	.002	1.03
General Impulsivity	0.28	0.14 – 0.42	< .001	2.97
Lack of Premeditation	0.08	-0.02 – 0.19	.119	1.69
Negative Urgency	0.08	-0.06 – 0.21	.264	3.06
Positive Urgency	0.25	0.12 – 0.38	<0.001	2.83
Sensation Seeking	0.02	-0.06 – 0.11	0.618	1.29

*Note*. VIF = Variance Inflation Factor.

**Table 5:** Regression Model 4 results predicting the antisocial facet.

Predictor	*β*	95% CI	*p*	*VIF*
Commission Errors	0.03	-0.05 – 0.10	.520	1.01
Decision Quality	-0.09	-0.18 – -0.00	.040	1.03
Delay Discounting	0.11	0.03 – 0.19	.007	1.03
General Impulsivity	0.02	-0.13 – 0.16	.827	2.97
Lack of Premeditation	0.06	-0.05 – 0.17	.284	1.69
Negative Urgency	0.02	-0.12 – 0.16	.763	3.06
Positive Urgency	0.38	0.25 – 0.52	< .001	2.83
Sensation Seeking	0.08	-0.01 – 0.17	.065	1.29

*Note*. VIF = Variance Inflation Factor.

Model 2 implemented the affective facet of psychopathy as the outcome variable. Model 2 (Table 3) revealed significant, positive associations among commission errors, delay discounting, and positive urgency. A significant negative association among the affective facet and decision quality also emerged. Model 2 accounted for 19% of the variability in the affective facet of psychopathy.

Model 3 implemented the lifestyle facet of psychopathy as the outcome variable. Model 3 (Table 4) revealed significant, positive associations among commission errors, delay discounting, general impulsivity, and positive urgency. Model 3 accounted for 33% of the variability in the lifestyle facet of psychopathy.

Model 4 implemented the antisocial facet of psychopathy as the outcome variable. Model 4 (Table 5) revealed significant, positive associations among delay discounting and positive urgency and the antisocial facet. Further, we also observed a negative association between the antisocial facet and decision quality. Model 4 accounted for 23% of the variability in the antisocial facet of psychopathy.

### Dominance analyses

To confirm the most important predictors of each facet of psychopathy we also conducted dominance analyses for each of our models. Our dominance analysis of Model 1 revealed that positive urgency was the single most important predictor as it exhibited complete dominance over all predictors (Table 6). Our bootstrap analyses suggested this finding was robust as it was replicated in 99% of our 5,000 bootstraps (S3 Table). Sensation seeking was the second-most important predictor, as it completely dominated all predictors except positive urgency. These findings replicated 71.83% of our bootstraps on average. Third was delay discounting due to its complete dominance over commission errors and lack of premeditation. These dominance results replicated in 70% of our bootstraps on average. Conversely, lack of premeditation and commission errors contributed 0% of the explained variability.

**Table 6:** Dominance matrix for Model 1 predicting the interpersonal facet arranged by order of complete dominance.

		1	2	3	4	5	6	7	8	R^2^	Rank
1	Positive Urgency	-	1	1	1	1	1	1	1	.09	1
2	Sensation Seeking		-	0.5	1	1	1	1	1	.03	2
3	Delay Discounting			-	0.5	0.5	0.5	1	1	.02	3
4	General Impulsivity				-	0.5	0.5	0.5	1	.01	4
5	Negative Urgency					-	0.5	0.5	1	.02	4
6	Decision Quality						-	1	0.5	.01	4
7	Commission Errors							-	0.5	.00	5
8	Lack of Premeditation								-	.00	5

*Notes*. A value of 1 indicates complete dominance of the row variable over the column variable, 0.5 indicates that complete dominance could not be established (e.g., conditional dominance) and a value of 0 indicated complete dominance of the column variable over the row variable. R^2^ indicates the average contribution of each predictor across all subset models.

**Fig 1:**
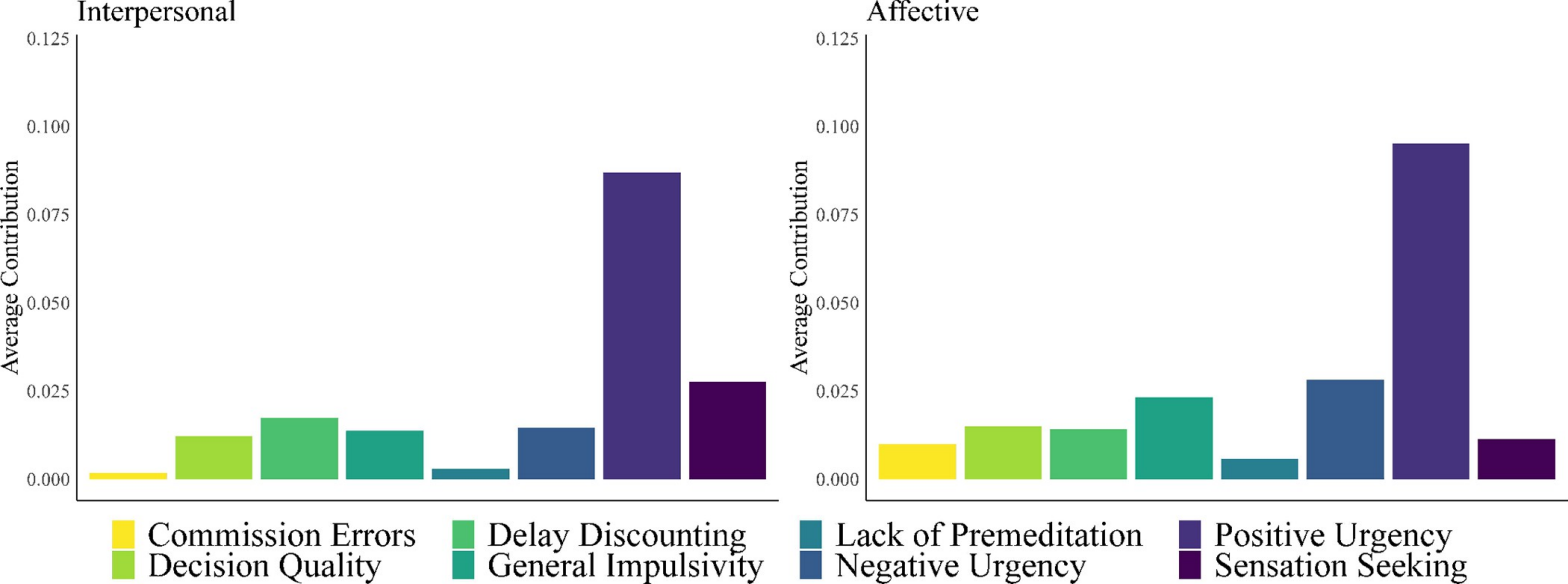
General dominance plots of the average contribution (via R^2^) of each predictor of the interpersonal (left) and affective (right) facets of psychopathy.

Our dominance analysis of Model 2 revealed that positive urgency completely dominated all other predictors of the affective facet (Table 7). As in Model 1, this dominance was highly stable as it was replicated in 99% of our bootstrap samples on average (S4 Table). No other instances of complete dominance were observed from Model 2. However, negative urgency contributed the second greatest amount of variance explained to the model, indicating it as the second-most dominant predictor from a general dominance (as opposed to complete dominance) perspective. General dominance values Models 1–2 are displayed in Fig 1.

**Table 7:** Dominance matrix for Model 2 predicting the affective facet arranged by order of complete dominance.

		1	2	3	4	5	6	7	8	R^2^	Rank
1	Positive Urgency	-	1	1	1	1	1	1	1	.10	1
2	General Impulsivity		-	0.5	0.5	0.5	0.5	0.5	0.5	.02	2
3	Negative Urgency			-	0.5	0.5	0.5	0.5	0.5	.03	2
4	Lack of Premeditation				-	0.5	0.5	0.5	0.5	.01	2
5	Sensation Seeking					-	0.5	0.5	0.5	.01	2
6	Decision Quality						-	0.5	0.5	.02	2
7	Delay Discounting							-	0.5	.01	2
8	Commission Errors								-	.01	2

*Notes*. A value of 1 indicates complete dominance of the row variable over the column variable, 0.5 indicates that complete dominance could not be established (e.g., conditional dominance) and a value of 0 indicated complete dominance of the column variable over the row variable. R^2^ indicates the average contribution of each predictor across all subset models.

Our dominance analysis of Model 3 revealed that positive urgency completely dominated all other predictors of the lifestyle facet excepting general impulsivity (Table 8). This dominance was largely stable, as it was replicated in 90% of our bootstraps on average (S5 Table). The second-most important predictor was the general impulsivity variable as it completely dominated lack of premeditation, sensation seeking, commission errors, and decision quality. The dominance of general impulsivity was also stable as these results replicated in 86.50% of our bootstraps. In terms of general dominance, commission errors and decision quality contributed very little to the variance explained by Model 3.

**Table 8:** Dominance matrix for Model 3 predicting the lifestyle facet arranged by order of complete dominance.

		1	2	3	4	5	6	7	8	R^2^	Rank
1	Positive Urgency	-	0.5	1	1	1	1	1	1	.11	1
2	General Impulsivity		-	0.5	0.5	1	1	1	1	.09	2
3	Delay Discounting			-	0.5	0.5	0.5	0.5	1	.02	3
4	Negative Urgency				-	0.5	0.5	0.5	0.5	.06	4
5	Lack of Premeditation					-	0.5	0.5	0.5	.03	4
6	Sensation Seeking						-	0.5	0.5	.02	4
7	Commission Errors							-	0.5	.01	4
8	Decision Quality								-	.01	4

*Notes*. A value of 1 indicates complete dominance of the row variable over the column variable, 0.5 indicates that complete dominance could not be established (e.g., conditional dominance) and a value of 0 indicated complete dominance of the column variable over the row variable. R^2^ indicates the average contribution of each predictor across all subset models.

**Fig 2:**
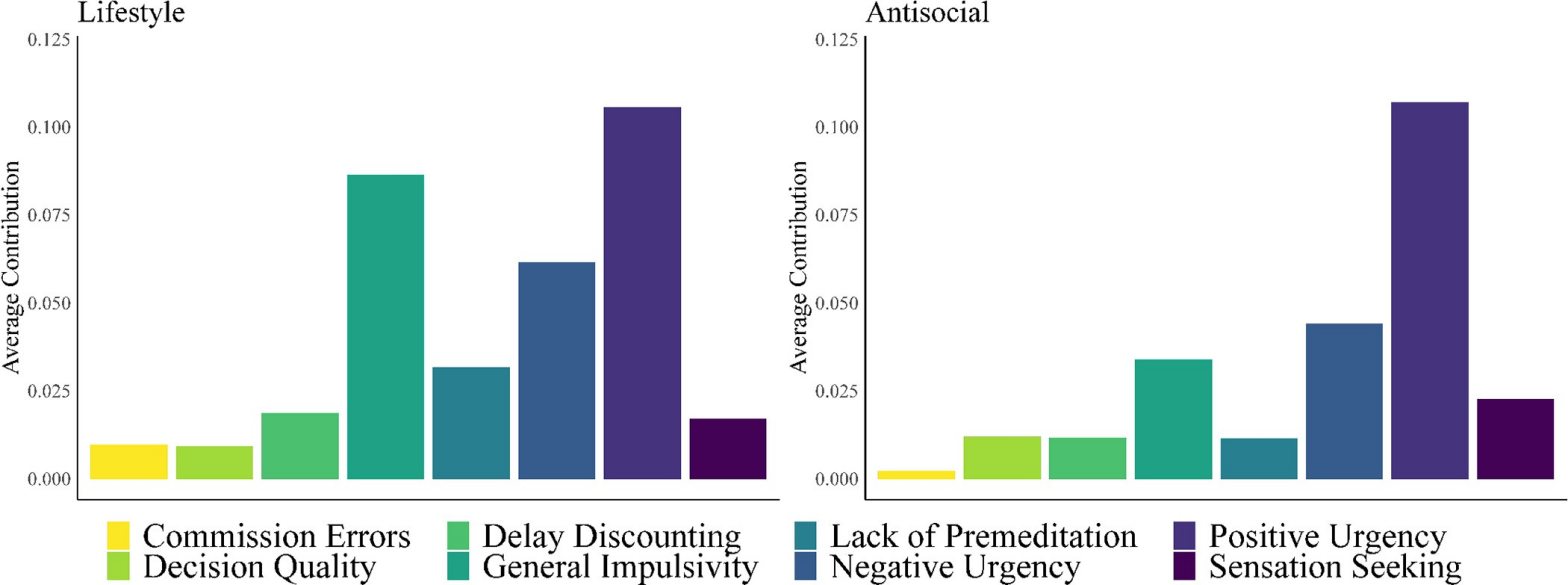
General dominance plots of the average contribution (via R^2^) of each predictor of the lifestyle (left) and antisocial (right) facets of psychopathy.

Our dominance analysis of Model 4 revealed that positive urgency completely dominated all other predictors of the antisocial facet (Table 9). The dominance of positive urgency was again found to be robust as it replicated in 98.57% of our bootstrap samples (S6 Table). The second-most important predictor was sensation seeking, as it completely dominated lack of premeditation, and commission errors as predictors of the antisocial facet. These results replicated in 64% of our bootstraps on average. In terms of general dominance, commission errors and decision quality contributed very little to the variance explained by Model 4. General dominance values from Models 3–4 are displayed in Fig 2.

**Table 9:** Dominance matrix for Model 4 predicting the antisocial facet arranged by order of complete dominance.

		1	2	3	4	5	6	7	8	R^2^	Rank
1	Positive Urgency	-	1	1	1	1	1	1	1	.11	1
2	Sensation Seeking		-	0.5	0.5	0.5	0.5	1	1	.02	2
3	Decision Quality			-	0.5	0.5	0.5	0.5	1	.01	3
4	Delay Discounting				-	0.5	0.5	0.5	1	.01	3
5	General Impulsivity					-	0.5	0.5	0.5	.03	4
6	Negative Urgency						-	0.5	0.5	.04	4
7	Lack of Premeditation							-	0.5	.01	4
8	Commission Errors								-	.01	4

*Notes*. A value of 1 indicates complete dominance of the row variable over the column variable, 0.5 indicates that complete dominance could not be established (e.g., conditional dominance) and a value of 0 indicated complete dominance of the column variable over the row variable. R^2^ indicates the average contribution of each predictor across all subset models.

### Multivariate model

To further contextualize our dominance findings from Models 1–4 we also conducted a multivariate dominance analysis. Multivariate dominance analysis treats the indicator variables as a single outcome. As such, this model reflects the overall impulsivity profile of psychopathy. Our multivariate dominance analysis relied on the *P*^*2*^ index of multivariate fit to test for complete dominance of predictors as per Azen & Budescu, 2006 [74]. Full model results are presented in S19 Table. Results from this model revealed positive urgency as the most important predictor of impulsivity as it showed complete dominance over all others excepting general impulsivity, for which complete dominance was not established. General impulsivity was the second most dominant variable in the multivariate model, followed by sensation seeking.

### Additional analyses

We initially included two additional measures of impulsivity in our analyses–false alarms from a Go/No-Go task and participant scores from the Iowa Gambling Task. However, we found that the inclusion of these variables contributed little to our models and artificially inflated the dominance values of other predictors. We excised these variables from our analyses to preserve power and to aid the interpretations of our dominance analyses. Results from these initial analyses largely mirrored the current analyses (see S7–S14 Tables). In addition, our sample included both substance dependent and non-substance dependent participants. In order to ensure none of our findings were impacted by the inclusion of both groups we included substance dependent group (i.e., 0 = non-dependent, 1 = dependent) as a covariate and examined possible group X impulsivity interactions. Only one such interaction emerged as significant for the affective facet of psychopathy between group and positive urgency, *p* = .038, but a simple slopes analysis indicated that the related slopes were not significant (S15–S18 Tables).

## Discussion

Psychopathy comprises a distinct set of traits and associated behaviors including interpersonal manipulation, shallow affect, erratic lifestyle, and antisocial behaviors. Psychopathy has repeatedly evinced positive links with various forms of impulsivity as assessed by a wide array of instruments (e.g., [34, 35, 39, 40]). In the current study we conducted a regimen of regression and dominance analyses using a curated set of impulsivity variables as predictors for each of the four facets of psychopathy to explore the potential for the psychopathy facets to reflect distinct profiles of impulsivity. We found that impulsivity stemming from positive affect (i.e., positive urgency) was the single most important form of impulsivity to all four facets of psychopathy and was the most important predictor in our combined multivariate model. We also found that response inhibition (i.e., commission errors) was the weakest correlate of psychopathy in all four models, consistent with recent meta-analytic work reflecting a weak association between psychopathy and response inhibition (e.g., *r* = -.14, [56]). Beyond these findings distinct profiles of impulsivity emerged for each of the facets.

### Profiles of impulsivity and psychopathy

Our findings suggest that there is indeed a strong link between psychopathy and impulsivity, but that this link, like the constructs themselves, is likely multifaceted. Our findings suggest that the interpersonal facet of psychopathy is marked by increased trait impulsivity by way of positive urgency and a tendency toward sensation seeking. At the neurobehavioral level the interpersonal facet was most strongly linked with temporal impulsivity, or a disregard for greater future rewards in light of lesser-immediate rewards. The affective facet was typified by affective impulsivity (via positive and negative urgency) and general impulsivity. Little to no variability in the affective facet was accounted for by the neurobehavioral components of impulsivity. The lifestyle facet yielded a similar profile of impulsivity to the affective facet with one exception: delay discounting rates accounted for more variance in the lifestyle facet. This difference suggests that the lifestyle facet may be better understood through the lens of intertemporal valuation of rewards. Finally, the antisocial facet was typified by positive urgency, sensation seeking, and negative urgency with the neurobehavioral components of impulsivity accounting for very little variance in this facet of psychopathy.

Some of our findings were inconsistent with prior studies, but not others. For example, prior research suggests that the Factor 2 psychopathy elements (lifestyle and antisocial facets) are positively linked with neurobehavioral impulsivity (e.g., [37, 59, 60]), whereas other work indicates that Factor 1 psychopathy (interpersonal and affective facets) is *negatively* linked with such impulsivity, acting as a buffer of sorts [61, 62]. However, the current findings suggest this may not be the case, as we observed *positive* links between both Factor 1 facets and delay discounting rates as well as a negative link between the affective facet and decision quality.

### Psychopathy and neurobehavioral aspects of impulsivity

In general, our neurobehavioral assessments shared very little variance with the four facets of psychopathy after the trait measures were accounted for. Of the neurobehavioral measures employed in this investigation only one appeared to be of importance to psychopathy: delay discounting. Although delay discounting was only identified as a dominant component of impulsivity for the interpersonal facet, it was also the only neurobehavioral measure in the current study that evinced significant, although modest, associations with all four facets of psychopathy in our initial regression models. This finding is consistent with research indicating that high delay discounters and psychopaths are at risk for similar externalizing behaviors (e.g., drug abuse; aggressive behavior [77, 78]). In contrast, commission errors and decision quality were both generally unreliable correlates of psychopathy. These findings suggest that the most relevant neurobehavioral dimension of impulsivity to psychopathy is temporal impulsivity (i.e., delay discounting). In this view, those high in psychopathy may utilize different valuation processes in respect to intertemporal choice, which may manifest as a disregard for larger-but-delayed rewards or a valuation bias in favor of smaller-but-immediate rewards. Further, it seems unlikely that response inhibition (i.e., commission errors) is responsible for much of the externalizing behaviors observed among psychopaths and that decision quality may only play a meaningful role among those with higher levels of the affective facet of psychopathy.

### Implications for psychopathy as a construct

The current study yielded evidence in support of traditional conceptualizations of psychopathy and its links to impulsivity (e.g., [32]). However, the current work also reveals a varied profile of impulsivity across each of the four facets. Our findings suggest that positive urgency is the primary component of impulsivity that is shared across all facets of psychopathy. This finding is consistent with the existing literature indicating that the factors of psychopathy yield similar, positive associations with positive urgency and that total psychopathy scores are more strongly associated with positive rather than negative urgency [43, 48]. One possible reason for this finding is the relative novelty of positive affect among those high in psychopathy as those high in psychopathy experience significantly elevated levels of negative affect and less subjective well-being (e.g., [79]). Indeed, novel experiences tend to evoke impulsive behavioral responses in humans and animals [80, 81].

Beyond positive urgency, we found that the impulsivity profiles of the facets were more similar across factors than within. Specifically, we observed that sensation seeking was a highly important component of impulsivity to the interpersonal (Factor 1) and antisocial (Factor 2) facets, but not the affective or lifestyle facets. We also found that the affective (Factor 1) and lifestyle (Factor 2) facets shared similar profiles of impulsivity, typified by positive urgency, general impulsivity and negative urgency. In practical terms these findings suggest certain impulsive actions are more likely in the context of psychopathic typologies. For example, a person high in the interpersonal facet may proactively seek out others to manipulate in pursuit of an elevated degree of arousal due to the importance of sensation-seeking to this facet. The positive experience of such arousal may then yield greater impulsive actions due to the involvement of positive urgency.

Temporal impulsivity was primarily important to the interpersonal facet, suggesting that this may be a compounding factor leading those higher in the interpersonal facet to seek lesser-but-immediate stimulation. Our interpretation of these findings is consistent with work demonstrating that those greater in psychopathy demonstrate altered patterns of functional connectivity among regions involved in subjective valuation (i.e., the striatum) and impulse control (i.e., the prefrontal cortex) which may underlie temporal discounting among those high in psychopathy [82]. In the context of intertemporal choice, portions of the prefrontal cortex inversely encode the deleterious effects of delay on the value of a given reward (that is, activity in these regions is negatively linked with reward delay) [83, 84]. As such, those with greater levels of the interpersonal facet may exhibit impulsivity due to a neural over-valuation of certain immediate rewards. However, we note that much of the literature testing the association between psychopathy and delay discounting has relied on domain-level measures of psychopathy rather than factor or facet-level scores [85]. In contrast, delay discounting was one of the least important predictors of the antisocial facet, being replaced by negative urgency. This finding is broadly consistent with the theoretical construction of the factor 2 traits as being highly anxious and work demonstrating that factor 2 is linked with greater negative emotion responses [86, 87]. Taken together, these findings suggest that affective impulsivity (i.e., positive and negative urgency) and boredom-related impulsivity (i.e., sensation seeking) comprise the antisocial facet’s links to impulsivity, whereas psychological valuation processes may be more important to the interpersonal facet than negative affect. Despite these patterns of dominance, we note here that delay discounting was a significant correlate of all four facets in our regression models. Regarding the affective and lifestyle facets, we found identical dominance profiles for these facets suggesting that the impulsivity of each facet is best described by general impulsiveness and affective impulsivity.

### Limitations

The findings of our study must be considered in light of several important limitations. First, our analyses are correlational and exploratory, relying entirely on cross-sectional data. As such, the results of our analyses should be considered preliminary and future work should attempt to confirm these findings with purpose-built studies. Our measure of delay discounting only examined monetary rewards. Although this is the most common application of this framework, recent research has revealed that provoked aggressive behavior is itself subjected to delay discounting, but that more antagonistic individuals show lesser discounting of aggression (e.g., [88, 89]). Future work should thus examine how intertemporal choices for such behaviors are related to psychopathy in contrast to monetary rewards. Similarly, the current study relied on a single measure of psychopathy. Recent work indicates that psychopathy may reflect divergent psychometric properties based on the modality of measurement used (i.e., structured interviews vs. self-reports; [90]). As such, our findings should also be replicated using different measures of psychopathy that utilize the four-facet model implemented here. Next, the current study occurred in Bulgaria and thus our findings could have been impacted by cultural considerations we were unable to control for in the current work. Indeed, research on psychopathy has primarily occurred in North America and Western Europe. Future studies should address the cross-cultural replicability of the findings presented here. Next, many of the forms of impulsivity in our analyses accounted for little variability (e.g., 3%) in the psychopathy facets. This was due in part to the nature of our analyses wherein we utilized multiple predictors that represented various aspects of a unifying construct. Future work is needed to better understand how such associations may translate to behavioral differences in naturalistic settings. Finally, our sample comprised a slight majority (55.33%) of abstinent drug users. This feature of our sample could have impacted some of our findings, as individuals who have successfully abstained from drug use may constitute a distinct set of individuals in relation to impulsivity. We thus urge caution in the generalization of our findings, though this concern is mitigated to some extent by the fact that the other 44.67% of our sample comprised those who had never used illicit substances and our supplemental analyses indicating there was no impact of group membership in the current findings. Future work should examine the profiles of impulsivity in current substance users and other samples to confirm our findings.

## Supporting information

S1 File Participants description. (PDF)

S1 Table Zero-order bivariate correlations of all study variables. * p < .05, ** p < .01, ***p < .001.
(PDF)

S2 Table Interrater reliability of the PCL:SV items. ASB = Antisocial Behavior, ICC = Intraclass Correlation Coefficient, LCI = Lower bound of the 95% confidence interval, UCI = Upper bound of the 95% confidence interval.
(PDF)

S3 Table Dominance analysis and bootstrap results predicting the interpersonal facet. D_ab_ = Original dominance analysis result, 1 indicates complete dominance of a over b, 0.5 indicates no dominance for either variable, 0 indicates complete dominance of b over a; M_Dab_ = mean dominance value from the 5,000 sample bootstrap procedure; P_ab_ = proportion of bootstraps where a completely dominated b; P_ba_ = proportion of bootstraps where b completely dominated a; P_nd_ = proportion of bootstraps that found no dominance.
(PDF)

S4 Table Dominance analysis and bootstrap results predicting the affective facet. D_ab_ = Original dominance analysis result, 1 indicates complete dominance of a over b, 0.5 indicates no dominance for either variable, 0 indicates complete dominance of b over a; M_Dab_ = mean dominance value from the 5,000 sample bootstrap procedure; P_ab_ = proportion of bootstraps where a completely dominated b; P_ba_ = proportion of bootstraps where b completely dominated a; P_nd_ = proportion of bootstraps that found no dominance.
(PDF)

S5 Table Dominance analysis and bootstrap results predicting the lifestyle facet. D_ab_ = Original dominance analysis result, 1 indicates complete dominance of a over b, 0.5 indicates no dominance for either variable, 0 indicates complete dominance of b over a; M_Dab_ = mean dominance value from the 5,000 sample bootstrap procedure; P_ab_ = proportion of bootstraps where a completely dominated b; P_ba_ = proportion of bootstraps where b completely dominated a; P_nd_ = proportion of bootstraps that found no dominance.
(PDF)

S6 Table Dominance analysis and bootstrap results predicting the antisocial facet. D_ab_ = Original dominance analysis result, 1 indicates complete dominance of a over b, 0.5 indicates no dominance for either variable, 0 indicates complete dominance of b over a; M_Dab_ = mean dominance value from the 5,000 sample bootstrap procedure; P_ab_ = proportion of bootstraps where a completely dominated b; P_ba_ = proportion of bootstraps where b completely dominated a; P_nd_ = proportion of bootstraps that found no dominance.
(PDF)

S7 Table Multiple regression model predicting the interpersonal facet of psychopathy. (PDF)

S8 Table Dominance analysis results predicting the interpersonal facet of psychopathy. Values of ‘1’ indicate complete dominance of the row variable over the column variable. Values of ‘0.5’ indicate that complete dominance could not be established.
(PDF)

S9 Table Multiple regression model predicting the affective facet of psychopathy. (PDF)

S10 Table Dominance analysis results predicting the affective facet of psychopathy. Values of ‘1’ indicate complete dominance of the row variable over the column variable. Values of ‘0.5’ indicate that complete dominance could not be established.
(PDF)

S11 Table Multiple regression model predicting the lifestyle facet of psychopathy. (PDF)

S12 Table Dominance analysis results predicting the lifestyle facet of psychopathy. Values of ‘1’ indicate complete dominance of the row variable over the column variable. Values of ‘0.5’ indicate that complete dominance could not be established.
(PDF)

S13 Table Multiple regression model predicting the antisocial facet of psychopathy. (PDF)

S14 Table Dominance analysis results predicting the antisocial facet of psychopathy. Values of ‘1’ indicate complete dominance of the row variable over the column variable. Values of ‘0.5’ indicate that complete dominance could not be established.
(PDF)

S15 Table Multiple regression model including group interactions predicting the interpersonal facet of psychopathy. Group indicates drug dependence such that 0 = non-dependent, 1 = dependent.
(PDF)

S16 Table Multiple regression model including group interactions predicting the affective facet of psychopathy. Group indicates drug dependence such that 0 = non-dependent, 1 = dependent.
(PDF)

S17 Table Multiple regression model including group interactions predicting the lifestyle facet of psychopathy. Group indicates drug dependence such that 0 = non-dependent, 1 = dependent.
(PDF)

S18 Table Multiple regression model including group interactions predicting the antisocial facet of psychopathy. Group indicates drug dependence such that 0 = non-dependent, 1 = dependent.
(PDF)

S19 Table Multivariate dominance analysis results predicting all facets of psychopathy. Values of ‘1’ indicate complete dominance of the row variable over the column variable. Values of ‘0.5’ indicate that complete dominance could not be established.
(PDF)

## References

[1] Neumann CS, Hare RD, Newman JP. The super-ordinate nature of the psychopathy checklist-revised. J Pers Disord. 2007;21(2):102–17. doi: 10.1521/pedi.2007.21.2.102 17492916 PMC3136810

[2] Ahn WY, Vassileva J. Machine-learning identifies substance-specific behavioral markers for opiate and stimulant dependence. Drug Alcohol Depend. 2016;161:247–57. doi: 10.1016/j.drugalcdep.2016.02.008 26905209 PMC4955649

[3] Vassileva J, Conrod PJ. Impulsivities and addictions: a multidimensional integrative framework informing assessment and interventions for substance use disorders. Philos Trans R Soc Lond B Biol Sci. 2019;374(1766):20180137. doi: 10.1098/rstb.2018.0137 30966920 PMC6335463

[4] Berger K, Rotermund P, Vieth ER, Hohnhorst A. The prognostic value of the PCL-R in relation to the SUD treatment ending. Int J Law Psychiatry. 2012;35(3):198–201. doi: 10.1016/j.ijlp.2012.02.008 22425292

[5] Salekin RT, Worley C, Grimes RD. Treatment of psychopathy: a review and brief introduction to the mental model approach for psychopathy. Behav Sci Law. 2010;28(2):235–66. doi: 10.1002/bsl.928 20422648

[6] Sewall LA, Olver ME. Psychopathy and treatment outcome: Results from a sexual violence reduction program. Personal Disord. 2019;10(1):59–69. doi: 10.1037/per0000297 29927298

[7] Hare RD. Manual for the Hare Psychopathy Checklist–Revised, 2nd edition. 2nd edition ed. Toronto: Multi-Health Systems; 2003.

[8] Forth AE, Kosson DS, Hare RD. The psychopathy checklist: Youth version (PCL: YV). Toronto, Ontario, Canada: Multi-Health Systems; 2003.

[9] Hart SD, Cox DN, Hare RD. Hare psychopathy checklist: Screening version (PCL:SV). Toronto: Multi-Heath Systems; 1995.

[10] Hare RD, Harpur TJ, Hakstian R, Forth AE, Hart SD, Newman JP. The revised psychopathy checklist: Reliability and factor structure. Psychological Assessment. 1990;2(3):338–41.

[11] Hare RD, Neumann CS. Psychopathy as a clinical and empirical construct. Annu Rev Clin Psychol. 2008;4:217–46. doi: 10.1146/annurev.clinpsy.3.022806.091452 18370617

[12] Neumann CS, Kosson DS, Forth AE, Hare RD. Factor structure of the Hare Psychopathy Checklist: Youth Version (PCL: YV) in incarcerated adolescents. Psychol Assess. 2006;18(2):142–54. doi: 10.1037/1040-3590.18.2.142 16768590

[13] Vitacco MJ, Neumann CS, Jackson RL. Testing a four-factor model of psychopathy and its association with ethnicity, gender, intelligence, and violence. J Consult Clin Psychol. 2005;73(3):466–76. doi: 10.1037/0022-006X.73.3.466 15982144

[14] Cunha O, Braga T, Goncalves RA. Psychopathy and Intimate Partner Violence. J Interpers Violence. 2021;36(3–4):NP1720–38NP. doi: 10.1177/0886260518754870 29366397

[15] Thomson ND, Bozgunov K, Psederska E, Vassileva J. Sex differences on the four-facet model of psychopathy predict physical, verbal, and indirect aggression. Aggress Behav. 2019;45(3):265–74. doi: 10.1002/ab.21816 30699249 PMC9036955

[16] Thomson ND, Vassileva J, Kiehl KA, Reidy D, Aboutanos M, McDougle R, et al. Which features of psychopathy and impulsivity matter most for prison violence? New evidence among female prisoners. Int J Law Psychiatry. 2019;64:26–33. doi: 10.1016/j.ijlp.2019.01.001 31122637

[17] Thomson ND. Psychopathy, the Four Facet Model, and Fearlessness: Testing Sympathetic and Parasympathetic Nervous System Reactivity in a Late Adolescent Sample. Journal of Psychopathology and Behavioral Assessment. 2022;44(1):51–63.

[18] West SJ, & Chester DS. The tangled webs we wreak: Examining the structure of aggressive personality using psychometric networks. Journal of Personality. 2022;90(5):762–80. doi: 10.1111/jopy.12695 34919275 PMC9203597

[19] Moeller FG, Barratt ES, Dougherty DM, Schmitz JM, Swann AC. Psychiatric aspects of impulsivity. Am J Psychiatry. 2001;158(11):1783–93. doi: 10.1176/appi.ajp.158.11.1783 11691682

[20] Patton JH, Stanford MS, Barratt ES. Factor structure of the Barratt impulsiveness scale. J Clin Psychol. 1995;51(6):768–74. doi: 10.1002/1097-4679(199511)51:6&lt;768::aid-jclp2270510607&gt;3.0.co;2-1 8778124

[21] Whiteside SP, Lynam DR. The five factor model and impulsivity: Using a structural model of personality to understand impulsivity. Personality and Individual Differences. 2001;30(4):669–89.

[22] Cyders MA, Coskunpinar A. Measurement of constructs using self-report and behavioral lab tasks: is there overlap in nomothetic span and construct representation for impulsivity? Clin Psychol Rev. 2011;31(6):965–82. doi: 10.1016/j.cpr.2011.06.001 21733491

[23] Hamilton KR, Mitchell MR, Wing VC, Balodis IM, Bickel WK, Fillmore M, et al. Choice impulsivity: Definitions, measurement issues, and clinical implications. Personal Disord. 2015;6(2):182–98. doi: 10.1037/per0000099 25867841 PMC4535726

[24] Kirby KN, Petry NM, Bickel WK. Heroin addicts have higher discount rates for delayed rewards than non-drug-using controls. J Exp Psychol Gen. 1999;128(1):78–87. doi: 10.1037//0096-3445.128.1.78 10100392

[25] Bechara A, Damasio AR, Damasio H, Anderson SW. Insensitivity to future consequences following damage to human prefrontal cortex. Cognition. 1994;50(1–3):7–15. doi: 10.1016/0010-0277(94)90018-3 8039375

[26] Rogers RD, Everitt BJ, Baldacchino A, Blackshaw AJ, Swainson R, Wynne K, et al. Dissociable deficits in the decision-making cognition of chronic amphetamine abusers, opiate abusers, patients with focal damage to prefrontal cortex, and tryptophan-depleted normal volunteers: evidence for monoaminergic mechanisms. Neuropsychopharmacology. 1999;20(4):322–39. doi: 10.1016/S0893-133X(98)00091-8 10088133

[27] Hamilton KR, Littlefield AK, Anastasio NC, Cunningham KA, Fink LHL, Wing VC, et al. Rapid-response impulsivity: definitions, measurement issues, and clinical implications. Personal Disord. 2015;6(2):168–81. doi: 10.1037/per0000100 25867840 PMC4476624

[28] Dougherty DM, Marsh DM, Mathias CW. Immediate and delayed memory tasks: a computerized behavioral measure of memory, attention, and impulsivity. Behav Res Methods Instrum Comput. 2002;34(3):391–8. doi: 10.3758/bf03195467 12395555

[29] Lane SD, Moeller FG, Steinberg JL, Buzby M, Kosten TR. Performance of cocaine dependent individuals and controls on a response inhibition task with varying levels of difficulty. Am J Drug Alcohol Abuse. 2007;33(5):717–26. doi: 10.1080/00952990701522724 17891664

[30] Allom V, Panetta G, Mullan B, Hagger MS. Self-report and behavioural approaches to the measurement of self-control: Are we assessing the same construct?. Personality and Individual Differences. 2016;90:137–42.

[31] Wennerhold L, Friese M, Vazire S. Why self-report measures of self-control and inhibition tasks do not substantially correlate. Collabra: Psychology. 2020;6(1):9.

[32] Hart SD, Dempster RJ. Impulsivity and psychopathy. In: Webster CD, Jackson MA, editors. Impulsivity: Theory, assessment, and treatment: The Guilford Press; 1997. p. 212–32.

[33] Edens JF, McDermott BE. Examining the construct validity of the Psychopathic Personality Inventory-Revised: preferential correlates of fearless dominance and self-centered impulsivity. Psychol Assess. 2010;22(1):32–42. doi: 10.1037/a0018220 20230149

[34] Poythress NG, Hall JR. Psychopathy and impulsivity reconsidered. Aggression and Violent Behavior,. 2011;16(2):120–34.

[35] Ray JV, Poythress NG, Weir JM, Rickelm A. Relationships between psychopathy and impulsivity in the domain of self-reported personality features. Personality and Individual Differences. 2009;46(2):83–7.

[36] Snowden RJ, Gray NS. Impulsivity and psychopathy: associations between the barrett impulsivity scale and the psychopathy checklist revised. Psychiatry Res. 2011;187(3):414–7. doi: 10.1016/j.psychres.2011.02.003 21377739

[37] Beszterczey S, Nestor PG, Shirai A, Harding S. Neuropsychology of decision making and psychopathy in high-risk ex-offenders. Neuropsychology. 2013;27(4):491–7. doi: 10.1037/a0033162 23876121

[38] Boulanger C, Habib M, LanAon C. Impaired making-decision and empathy disorder in psychopathy. In: Psychiatry E, editor. 16th AEP Congress 2008. p. S92.

[39] Kim YY, Jung YS. Reduced frontal activity during response inhibition in individuals with psychopathic traits: an sLORETA study. Biol Psychol. 2014;97:49–59. doi: 10.1016/j.biopsycho.2014.02.004 24553134

[40] Krakowski MI, Foxe J, de Sanctis P, Nolan K, Hoptman MJ, Shope C, et al. Aberrant response inhibition and task switching in psychopathic individuals. Psychiatry Res. 2015;229(3):1017–23. doi: 10.1016/j.psychres.2015.06.018 26257091

[41] Psederska E, Thomson ND, Bozgunov K, Nedelchev D, Vasilev G, Vassileva J. Effects of Psychopathy on Neurocognitive Domains of Impulsivity in Abstinent Opiate and Stimulant Users. Front Psychiatry. 2021;12:660810. doi: 10.3389/fpsyt.2021.660810 34177649 PMC8219927

[42] Vassileva J, Petkova P, Georgiev S, Martin EM, Tersiyski R, Raycheva M, et al. Impaired decision-making in psychopathic heroin addicts. Drug Alcohol Depend. 2007;86(2–3):287–9. doi: 10.1016/j.drugalcdep.2006.06.015 16930861

[43] Gray NS, Weidacker K, Snowden RJ. Psychopathy and impulsivity: The relationship of psychopathy to different aspects of UPPS-P impulsivity. Psychiatry Res. 2019;272:474–82. doi: 10.1016/j.psychres.2018.12.155 30611967

[44] Morgan JE, Gray NS, Snowden RJ. The relationship between psychopathy and impulsivity: A multi-impulsivity measurement approach. Personality and Individual Differences. 2011;51(4):429–34.

[45] Anestis MD, Anestis JC, Joiner TE. Affective considerations in antisocial behavior: An examination of negative urgency in primary and secondary psychopathy. Personality and Individual Differences. 2009;47(6):668–70.

[46] Berg JM, Latzman RD, Bliwise NG, Lilienfeld SO. Parsing the heterogeneity of impulsivity: A meta-analytic review of the behavioral implications of the UPPS for psychopathology. Psychol Assess. 2015;27(4):1129–46. doi: 10.1037/pas0000111 25822833

[47] Hollerbach P, Habermeyer E, Nitschke J, Sünkel Z, Mokros A. Construct validity of the German version of the Hare psychopathy checklist–revised. European Journal of Psychological Assessment. 2020;36(5):805–16.

[48] Miller JD, Watts A, Jones SE. Does psychopathy manifest divergent relations with components of its nomological network depending on gender? Personality and Individual Differences. 2011;50(5):564–9.

[49] Weidacker K, O’Farrell KR, Gray NS, Johnston SJ, Snowden RJ. Psychopathy and impulsivity: The relationship of the triarchic model of psychopathy to different forms of impulsivity in offenders and community participants. Personality and Individual Differences. 2017;114:134–9.

[50] Blair KS, Morton J, Leonard A, Blair RJR. Impaired decision-making on the basis of both reward and punishment information in individuals with psychopathy. Personality and Individual Differences. 2006;41(1):155–65.

[51] Blair RJ, Colledge E, Mitchell DG. Somatic markers and response reversal: is there orbitofrontal cortex dysfunction in boys with psychopathic tendencies? J Abnorm Child Psychol. 2001;29(6):499–511. doi: 10.1023/a:1012277125119 11761284

[52] Mitchell DG, Colledge E, Leonard A, Blair RJ. Risky decisions and response reversal: is there evidence of orbitofrontal cortex dysfunction in psychopathic individuals? Neuropsychologia. 2002;40(12):2013–22. doi: 10.1016/s0028-3932(02)00056-8 12207998

[53] van Honk J, Hermans EJ, Putman P, Montagne B, Schutter DJ. Defective somatic markers in sub-clinical psychopathy. Neuroreport. 2002;13(8):1025–7. doi: 10.1097/00001756-200206120-00009 12060801

[54] Lapierre D, Braun CM, Hodgins S. Ventral frontal deficits in psychopathy: neuropsychological test findings. Neuropsychologia. 1995;33(2):139–51. doi: 10.1016/0028-3932(94)00110-b 7746360

[55] Roussy S, Toupin J. Behavioral inhibition deficits in juvenile psychopaths. Aggressive Behavior. 2000;26(6):413–24.

[56] Munro GE, Dywan J, Harris GT, McKee S, Unsal A, Segalowitz SJ. Response inhibition in psychopathy: the frontal N2 and P3. Neurosci Lett. 2007;418(2):149–53. doi: 10.1016/j.neulet.2007.03.017 17418489

[57] Vassileva J, Georgiev S, Martin E, Gonzalez R, Segala L. Psychopathic heroin addicts are not uniformly impaired across neurocognitive domains of impulsivity. Drug Alcohol Depend. 2011;114(2–3):194–200. doi: 10.1016/j.drugalcdep.2010.09.021 21112701 PMC3062675

[58] Verona E, Sprague J, Sadeh N. Inhibitory control and negative emotional processing in psychopathy and antisocial personality disorder. J Abnorm Psychol. 2012;121(2):498–510. doi: 10.1037/a0025308 22288907

[59] Dean AC, Altstein LL, Berman ME, Constans JI, Sugar CA, McCloskey MS. Secondary Psychopathy, but not Primary Psychopathy, is Associated with Risky Decision-Making in Noninstitutionalized Young Adults. Pers Individ Dif. 2013;54(2):272–7. doi: 10.1016/j.paid.2012.09.009 23185100 PMC3505104

[60] Miranda R Jr., MacKillop J, Meyerson LA, Justus A, Lovsallo WR. Influence of antisocial and psychopathic traits on decision-making biases in alcoholics. Alcohol Clin Exp Res. 2009;33(5):817–25. doi: 10.1111/j.1530-0277.2009.00901.x 19298325 PMC2846168

[61] Feilhauer J, Cima M, Korebrits A, Kunert HJ. Differential associations between psychopathy dimensions, types of aggression, and response inhibition. Aggress Behav. 2012;38(1):77–88. doi: 10.1002/ab.20415 22028178

[62] Weidacker K, Snowden RJ, Boy F, Johnston SJ. Response inhibition in the parametric Go/No-Go task in psychopathic offenders. Psychiatry Res. 2017;250:256–63. doi: 10.1016/j.psychres.2017.01.083 28171793

[63] Wilson MJ, Abramowitz C, Vasilev G, Bozgunov K, Vassileva J. Psychopathy in Bulgaria: The cross-cultural generalizability of the Hare Psychopathy Checklist. J Psychopathol Behav Assess. 2014;36(3):389–400. doi: 10.1007/s10862-014-9405-6 25313268 PMC4193952

[64] Veal R, Critchley C, Luebbers S, Cossar R, Ogloff JR. Factor structure of the Psychopathy Checklist: Screening Version (PCL:SV): A systematic review using narrative synthesis. Journal of Psychopathology and Behavioral Assessment. 2021;43(3):565–82.

[65] Lynam DR, Smith GT, Whiteside SP, Cyders MA. The UPPS-P: Assessing five personality pathways to impulsive behavior (technical report). West Lafayette, IN: Purdue University; 2006.

[66] Zuckerman M, Eysenck S, Eysenck HJ. Sensation seeking in England and America: cross-cultural, age, and sex comparisons. J Consult Clin Psychol. 1978;46(1):139–49. doi: 10.1037//0022-006x.46.1.139 627648

[67] Vitacco MJ, Rogers R. Predictors of adolescent psychopathy: the role of impulsivity, hyperactivity, and sensation seeking. J Am Acad Psychiatry Law. 2001;29(4):374–82. 11785608

[68] De Brito SA, Viding E, Kumari V, Blackwood N, Hodgins S. Cool and hot executive function impairments in violent offenders with antisocial personality disorder with and without psychopathy. PLoS One. 2013;8(6):e65566. doi: 10.1371/journal.pone.0065566 23840340 PMC3688734

[69] Swann AC, Lijffijt M, Lane SD, Steinberg JL, Moeller FG. Trait impulsivity and response inhibition in antisocial personality disorder. J Psychiatr Res. 2009;43(12):1057–63. doi: 10.1016/j.jpsychires.2009.03.003 19345957 PMC2716408

[70] Kvam PD, Romeu RJ, Turner BM, Vassileva J, Busemeyer JR. Testing the factor structure underlying behavior using joint cognitive models: Impulsivity in delay discounting and Cambridge gambling tasks. Psychol Methods. 2021;26(1):18–37. doi: 10.1037/met0000264 32134313 PMC7483167

[71] Raven J. The Raven’s progressive matrices: change and stability over culture and time. Cogn Psychol. 2000;41(1):1–48. doi: 10.1006/cogp.1999.0735 10945921

[72] Gamer M, Lemon J, Singh I. irr: Various coefficients of interrater reliability and agreement. R package version 0.84.1. https://cran.r-project.org/web/packages/irr/2019.

[73] Azen R, Budescu DV. The dominance analysis approach for comparing predictors in multiple regression. Psychological Methods. 2003;8(2):129–48. doi: 10.1037/1082-989x.8.2.129 12924811

[74] Azen R, Budescu DV. Comparing predictors in multivariate regression models: An extension of dominance analysis. Journal of Educational and Behavioral Statistics. 2006;31(2):157–80.

[75] Navarrete CB, Soares C. dominanceAnalysis: Dominance analysis for general, generalized and mixed linear models. R package version 2.00. https://cran.r-project.org/web/packages/dominanceanalysis2020.

[76] Blais J, Forth AE, Hare RD. Examining the interrater reliability of the Hare Psychopathy Checklist-Revised across a large sample of trained raters. Psychol Assess. 2017;29(6):762–75. doi: 10.1037/pas0000455 28594218

[77] Koepfler J, Brewster J, Stoloff M, Saville B. Predicting police aggression: Comparing traditional and non-traditional prediction models. Journal of Police and Criminal Psychology. 2012;27(2):141–9.

[78] Reynolds B. A review of delay-discounting research with humans: relations to drug use and gambling. Behav Pharmacol. 2006;17(8):651–67. doi: 10.1097/FBP.0b013e3280115f99 17110792

[79] Love AB, Holder MD. Psychopathy and subjective well-being. Personality and Individual Differences. 2014;66:112–7.

[80] Wang MZ, Marshall AT, Kirkpatrick K. Differential effects of social and novelty enrichment on individual differences in impulsivity and behavioral flexibility. Behav Brain Res. 2017;327:54–64. doi: 10.1016/j.bbr.2017.03.028 28341610 PMC5473952

[81] Wood AC, Rijsdijk F, Asherson P, Kuntsi J. Inferring Causation from Cross-Sectional Data: Examination of the Causal Relationship between Hyperactivity-Impulsivity and Novelty Seeking. Front Genet. 2011;2:6. doi: 10.3389/fgene.2011.00006 22303305 PMC3268378

[82] Hosking JG, Kastman EK, Dorfman HM, Samanez-Larkin GR, Baskin-Sommers A, Kiehl KA, et al. Disrupted Prefrontal Regulation of Striatal Subjective Value Signals in Psychopathy. Neuron. 2017;95(1):221–31 e4. doi: 10.1016/j.neuron.2017.06.030 28683266 PMC5796650

[83] Ballard K, Knutson B. Dissociable neural representations of future reward magnitude and delay during temporal discounting. Neuroimage. 2009;45(1):143–50. doi: 10.1016/j.neuroimage.2008.11.004 19071223 PMC2685201

[84] Li N, Ma N, Liu Y, He XS, Sun DL, Fu XM, et al. Resting-state functional connectivity predicts impulsivity in economic decision-making. J Neurosci. 2013;33(11):4886–95. doi: 10.1523/JNEUROSCI.1342-12.2013 23486959 PMC6618998

[85] Malesza M, & Kalinowski K. Willingness to share, impulsivity and the Dark Triad traits. Current Psychology. 2021;40:3888–96.

[86] Skeem JL, Poythress N., Edens J. F., Lilienfeld S. O., & Cale E. M. Psychopathic personality or personalities? Exploring potential variants of psychopathy and their implications for risk assessment. Aggression and Violent Behavior,. 2003;8(5):513–46.

[87] Kimonis ER, Frick PJ, Cauffman E, Goldweber A, Skeem J. Primary and secondary variants of juvenile psychopathy differ in emotional processing. Dev Psychopathol. 2012;24(3):1091–103. doi: 10.1017/S0954579412000557 22781873

[88] Chester DS, Bell SB, DeWall CN, West SJ, Romero-Lopez M, Craig AW. Neural correlates of intertemporal choice in aggressive behavior. Aggress Behav. 2019;45(5):507–16. doi: 10.1002/ab.21838 30989667 PMC7388593

[89] West SJ, Lasko EN, Hall CJ, Khan NG, Chester DS. Some revenge now or more revenge later? Applying an intertemporal framework to retaliatory aggression. Motivation Science. 2021;8(1):33–55.

[90] West SJ, Psederska E, Vasilev G, Bozgunov K, Nedelchev D, Thomson ND, et al. Comparing psychopathy across measurement modalities. Personal Disord. 2022. doi: 10.1037/per0000565 35446100 PMC11087072

