# Supplementary material for: Identifying distinct profiles of impulsivity for the four facets of psychopathy
Source: PLoS One. 2023 Apr 14;18(4):e0283866. doi: 10.1371/journal.pone.0283866 (PMC10104332; doi:10.1371/journal.pone.0283866)
Supplement: S1 File — (PDF) [file pone.0283866.s001.pdf]

S1 File. Participants description.

Participants were recruited from a larger study on impulsivity among substance users in Bulgaria via flyers placed at substance abuse clinics, therapeutic communities, social venues, as well as through the study's web page and Facebook page. Participants were initially screened via telephone on their medical and substance use histories. All participants had to meet the following inclusion criteria: (1) age between 18 and 50 years, (2) Raven's Progressive Matrices (101) estimated IQ higher than 75; (3) minimum of 8th grade education; (4) being able to read and write in Bulgarian; (5) HIV-seronegative status; (6) negative breathalyzer test for alcohol and negative urine toxicology screen for amphetamines, methamphetamines, cocaine, opiates, methadone, cannabis, benzodiazepines, barbiturates, and MDMA. Exclusion criteria included history of neurological illness, head injury with loss of consciousness of more than 30min, and history of psychotic disorders and/or use of antipsychotic medication.

The initial sample included 793 individuals (66.50% male), with a mean age of 28.96 years (SD = 7.24). A total of 450 participants (56.74%) had a history of substance dependence, of whom 140 were mono-dependent on heroin, 131 were mono-dependent on amphetamines and 179 were polysubstance dependent. Most of the study participants with a history of substance dependence (64.50%) were in protracted abstinence (more than 12 months of abstinence) at the time of testing.

We excluded participants who did not have complete data from any of our measures of interest (i.e., listwise deletion). This approach was necessary because the dominanceAnalysis package does not allow for missing data and multiple imputation of missing data is not an option when nonparametric bootstrapping procedures are needed. As such, our final sample comprised 479 participants in total. The demographics of this subset of participants closely mirrored those

of the full sample. Specifically, our subsample was 29.59 years of age on average ($SD = 7.46$), 64.70% male, and contained 266 participants with a history of substance dependence (55.53% of the subsample). Of these participants, 81 were mono-dependent on heroin, 79 were mono-dependent on amphetamines and 106 were polysubstance dependent.
