# Supplementary material for: Identifying distinct profiles of impulsivity for the four facets of psychopathy
Source: PLoS One. 2023 Apr 14;18(4):e0283866. doi: 10.1371/journal.pone.0283866 (PMC10104332; doi:10.1371/journal.pone.0283866)
Supplement: S1 Table — * p < .05, ** p < .01, ***p < .001. (PDF) [file pone.0283866.s002.pdf]

**S1 Table. Zero-order Bivariate Correlations of All Study Variables.**

| | | 1 | 2 | 3 | 4 | 5 | 6 | 7 | 8 | 9 | 10 | 11 |
|---|---|---|---|---|---|---|---|---|---|---|---|---|
| 1 | Affect | - | | | | | | | | | | |
| 2 | Antisocial | .74*** | - | | | | | | | | | |
| 3 | Lifestyle | .59*** | .73*** | - | | | | | | | | |
| 4 | General Impulsivity | .28*** | .34*** | .47*** | - | | | | | | | |
| 5 | Decision Quality | -.12** | -.12** | -.12** | -.10* | - | | | | | | |
| 6 | Delay Discounting | .13*** | .12*** | .15*** | -.01 | -.12* | - | | | | | |
| 7 | Commission Errors | .09* | .06 | .11** | .03 | -.06 | .07* | - | | | | |
| 8 | Interpersonal | .61*** | .58*** | .51*** | .21*** | -.09* | .13*** | .03 | - | | | |
| 9 | Lack of Premeditation | .16*** | .21*** | .31*** | .63*** | -.02 | -.05 | .03 | .11** | - | | |
| 10 | Negative Urgency | .29*** | .37*** | .44*** | .68*** | -.05 | -.04 | .04 | .20*** | .35*** | - | |
| 11 | Positive Urgency | .40*** | .46*** | .49*** | .63*** | -.08 | -.01 | .04 | .34*** | .34*** | .77*** | - |
| 12 | Sensation Seeking | .19*** | .25*** | .25*** | .40*** | -.03 | -.03 | -.02 | .22*** | .28*** | .29*** | .41*** |

*Note.* * $p < .05$, ** $p < .01$, *** $p < .001$.
