# Supplementary material for: Identifying distinct profiles of impulsivity for the four facets of psychopathy
Source: PLoS One. 2023 Apr 14;18(4):e0283866. doi: 10.1371/journal.pone.0283866 (PMC10104332; doi:10.1371/journal.pone.0283866)
Supplement: S2 Table — ASB = Antisocial Behavior, ICC = Intraclass Correlation Coefficient, LCI = Lower bound of the 95% confidence interval, UCI = Upper bound of the 95% confidence interval. (PDF) [file pone.0283866.s003.pdf]

S2 Table. Interrater Reliability of the PCL:SV Items.

Item	ICC	LCI	UCI
Interpersonal			
Superficial	0.71	0.62	0.78
Grandiose	0.81	0.75	0.86
Deceitful	0.84	0.78	0.88
Affect			
No Remorse	0.85	0.80	0.89
No Empathy	0.81	0.74	0.86
No Responsibility	0.81	0.75	0.86
Lifestyle			
Impulsive	0.89	0.85	0.92
No Goals	0.88	0.84	0.91
Irresponsible	0.78	0.70	0.83
Antisocial			
Poor Behavioral Control	0.88	0.84	0.91
Adolescent ASB	0.96	0.94	0.97
Adult ASB	0.94	0.92	0.96

Note. ASB = Antisocial Behavior, ICC = Intraclass Correlation Coefficient, LCI = Lower bound of the 95% confidence interval, UCI = Upper bound of the 95% confidence interval.
