# Supplementary material for: Identifying distinct profiles of impulsivity for the four facets of psychopathy
Source: PLoS One. 2023 Apr 14;18(4):e0283866. doi: 10.1371/journal.pone.0283866 (PMC10104332; doi:10.1371/journal.pone.0283866)
Supplement: S5 Table — Dab = Original dominance analysis result, 1 indicates complete dominance of a over b, 0.5 indicates no dominance for either variable, 0 indicates complete dominance of b over a; MDab = mean dominance value from the 5,000 sample bootstrap procedure; Pab = proportion of bootstraps where a completely dominated b; Pba = proportion of bootstraps where b completely dominated a; Pnd = proportion of bootstraps that found no dominance. (PDF) [file pone.0283866.s006.pdf]

S5 Table. Dominance Analysis and Bootstrap Results Predicting the Lifestyle Facet.

Variable a	Variable b	D_{ab}	M_{Dab}	SE	P_{ab}	P_{ba}	P_{nd}
General Impulsivity	Negative Urgency	0.5	0.68	0.24	0.35	0.00	0.65
General Impulsivity	Positive Urgency	0.5	0.33	0.26	0.02	0.36	0.62
General Impulsivity	Lack of Premeditation	1	0.91	0.19	0.82	0.00	0.18
General Impulsivity	Sensation Seeking	1	0.96	0.13	0.93	0.00	0.07
General Impulsivity	Decision Quality	1	0.79	0.25	0.57	0.00	0.43
General Impulsivity	Delay Discounting	0.5	0.68	0.24	0.35	0.00	0.65
General Impulsivity	Commission Errors	1	0.80	0.24	0.61	0.00	0.39
Negative Urgency	Positive Urgency	0	0.11	0.21	0.00	0.78	0.22
Negative Urgency	Lack of Premeditation	0.5	0.52	0.10	0.04	0.00	0.96
Negative Urgency	Sensation Seeking	0.5	0.61	0.21	0.23	0.00	0.77
Negative Urgency	Decision Quality	0.5	0.55	0.15	0.10	0.00	0.90
Negative Urgency	Delay Discounting	0.5	0.50	0.05	0.01	0.00	0.99
Negative Urgency	Commission Errors	0.5	0.51	0.07	0.02	0.00	0.98
Positive Urgency	Lack of Premeditation	1	0.87	0.22	0.75	0.00	0.25
Positive Urgency	Sensation Seeking	1	0.97	0.13	0.93	0.00	0.07
Positive Urgency	Decision Quality	1	0.94	0.17	0.88	0.00	0.12
Positive Urgency	Delay Discounting	1	0.89	0.21	0.78	0.00	0.22
Positive Urgency	Commission Errors	1	0.95	0.15	0.90	0.00	0.10
Lack of Premeditation	Sensation Seeking	0.5	0.52	0.10	0.04	0.00	0.96
Lack of Premeditation	Decision Quality	0.5	0.54	0.14	0.08	0.00	0.92
Lack of Premeditation	Delay Discounting	0.5	0.50	0.02	0.00	0.00	1.00
Lack of Premeditation	Commission Errors	0.5	0.51	0.07	0.02	0.00	0.98
Sensation Seeking	Decision Quality	0.5	0.54	0.13	0.07	0.00	0.93
Sensation Seeking	Delay Discounting	0.5	0.50	0.04	0.00	0.00	0.99
Sensation Seeking	Commission Errors	0.5	0.51	0.05	0.01	0.00	0.99
Decision Quality	Delay Discounting	0	0.36	0.27	0.05	0.33	0.62
Decision Quality	Commission Errors	0.5	0.49	0.24	0.10	0.12	0.78
Delay Discounting	Commission Errors	0.5	0.66	0.28	0.36	0.05	0.59

Notes. D_{ab} = Original dominance analysis result, 1 indicates complete dominance of a over b, 0.5 indicates no dominance for either variable, 0 indicates complete dominance of b over a; M_{Dab} = mean dominance value from the 5,000 sample bootstrap procedure; P_{ab} = proportion of bootstraps where a completely dominated b; P_{ba} = proportion of bootstraps where b completely dominated a; P_{nd} = proportion of bootstraps that found no dominance.
