# Supplementary material for: Identifying distinct profiles of impulsivity for the four facets of psychopathy
Source: PLoS One. 2023 Apr 14;18(4):e0283866. doi: 10.1371/journal.pone.0283866 (PMC10104332; doi:10.1371/journal.pone.0283866)
Supplement: S7 Table — (PDF) [file pone.0283866.s008.pdf]

S7 Table. Multiple Regression Model Predicting the Interpersonal Facet of Psychopathy.

Predictors	*Estimates*	*CI*	*p*
(Intercept)	0.01	-0.08 – 0.09	0.906
General Impulsivity	0.06	-0.08 – 0.21	0.385
Sensation Seeking	0.15	0.05 – 0.25	0.004
Negative Urgency	-0.14	-0.29 – 0.01	0.066
Positive Urgency	0.34	0.19 – 0.48	<0.001
Decision Quality	-0.10	-0.20 – -0.01	0.027
Delay Discounting	0.13	0.05 – 0.22	0.003
IGT total	-0.01	-0.10 – 0.08	0.781
False Alarms (GNG)	0.10	-0.10 – 0.30	0.325
Commission Errors	0.03	-0.05 – 0.12	0.455
Lack of Premeditation	-0.02	-0.14 – 0.09	0.675
