# Supplementary material for: Identifying distinct profiles of impulsivity for the four facets of psychopathy
Source: PLoS One. 2023 Apr 14;18(4):e0283866. doi: 10.1371/journal.pone.0283866 (PMC10104332; doi:10.1371/journal.pone.0283866)
Supplement: S9 Table — (PDF) [file pone.0283866.s010.pdf]

**S9 Table. Multiple Regression Model Predicting the Affective Facet of Psychopathy.**

| *Predictors* | *Estimates* | *CI* | *p* |
|---|---|---|---|
| (Intercept) | 0.01 | -0.08 – 0.09 | 0.873 |
| General Impulsivity | 0.11 | -0.04 – 0.25 | 0.142 |
| Sensation Seeking | 0.01 | -0.09 – 0.11 | 0.865 |
| Negative Urgency | -0.07 | -0.21 – 0.08 | 0.358 |
| Positive Urgency | 0.37 | 0.23 – 0.51 | <0.001 |
| Decision Quality | -0.11 | -0.20 – -0.02 | 0.019 |
| Delay Discounting | 0.11 | 0.03 – 0.20 | 0.011 |
| IGT total | 0.02 | -0.07 – 0.10 | 0.710 |
| False Alarms (GNG) | 0.13 | -0.07 – 0.32 | 0.199 |
| Commission Errors | 0.11 | 0.02 – 0.19 | 0.015 |
| Lack of Premeditation | -0.01 | -0.12 – 0.10 | 0.860 |
