# Supplementary material for: Identifying distinct profiles of impulsivity for the four facets of psychopathy
Source: PLoS One. 2023 Apr 14;18(4):e0283866. doi: 10.1371/journal.pone.0283866 (PMC10104332; doi:10.1371/journal.pone.0283866)
Supplement: S11 Table — (PDF) [file pone.0283866.s012.pdf]

S11 Table. Multiple Regression Model Predicting the Lifestyle Facet of Psychopathy.

Predictors	*Estimates*	*CI*	*p*
General Impulsivity	0.25	0.12 – 0.38	<0.001
Sensation Seeking	0.02	-0.06 – 0.11	0.587
Negative Urgency	0.07	-0.06 – 0.21	0.272
Positive Urgency	0.22	0.09 – 0.35	0.001
Decision Quality	-0.03	-0.11 – 0.06	0.530
Delay Discounting	0.12	0.04 – 0.20	0.003
IGT total	-0.08	-0.16 – -0.00	0.044
False Alarms (GNG)	0.24	0.06 – 0.42	0.008
Commission Errors	0.08	0.00 – 0.16	0.042
Lack of Premeditation	0.07	-0.03 – 0.17	0.166
