# Supplementary material for: Identifying distinct profiles of impulsivity for the four facets of psychopathy
Source: PLoS One. 2023 Apr 14;18(4):e0283866. doi: 10.1371/journal.pone.0283866 (PMC10104332; doi:10.1371/journal.pone.0283866)
Supplement: S12 Table — Values of ‘1’ indicate complete dominance of the row variable over the column variable. Values of ‘0.5’ indicate that complete dominance could not be established. (PDF) [file pone.0283866.s013.pdf]

S12 Table. Dominance Analysis Results Predicting the Lifestyle Facet of Psychopathy.

		1	2	3	4	5	6	7	8	9	10
1	General Impulsivity	-	0.5	0.5	1	1	1	1	1	1	1
2	Positive Urgency		-	1	1	1	0.5	1	1	1	1
3	Negative Urgency			-	0.5	0.5	0.5	0.5	0.5	1	0.5
4	False Alarms (GNG)				-	0.5	0.5	0.5	0.5	0.5	1
5	Commission Errors					-	0.5	0.5	0.5	0.5	1
6	Lack of Premeditation						-	0.5	0.5	0.5	0.5
7	Delay Discounting							-	0.5	0.5	0.5
8	IGT total								-	0.5	0.5
9	Sensation Seeking									-	0.5
10	Decision Quality										-

Notes. Values of ‘1’ indicates complete dominance of the row variable over the column variable.

Values of ‘0.5’ indicate that complete dominance could not be established.
