# Supplementary material for: Identifying distinct profiles of impulsivity for the four facets of psychopathy
Source: PLoS One. 2023 Apr 14;18(4):e0283866. doi: 10.1371/journal.pone.0283866 (PMC10104332; doi:10.1371/journal.pone.0283866)
Supplement: S13 Table — (PDF) [file pone.0283866.s014.pdf]

S13 Table. Multiple Regression Model Predicting the Antisocial Facet of Psychopathy.

Predictors	*Estimates*	*CI*	*p*
General Impulsivity	0.04	-0.10 – 0.18	0.585
Sensation Seeking	0.06	-0.03 – 0.16	0.192
Negative Urgency	0.04	-0.10 – 0.18	0.549
Positive Urgency	0.35	0.21 – 0.48	<0.001
Decision Quality	-0.07	-0.16 – 0.01	0.099
Delay Discounting	0.10	0.02 – 0.19	0.017
IGT total	-0.06	-0.14 – 0.02	0.156
False Alarms (GNG)	0.20	0.01 – 0.39	0.035
Commission Errors	0.03	-0.06 – 0.11	0.543
Lack of Premeditation	0.05	-0.06 – 0.16	0.367
