# Supplementary material for: Identifying distinct profiles of impulsivity for the four facets of psychopathy
Source: PLoS One. 2023 Apr 14;18(4):e0283866. doi: 10.1371/journal.pone.0283866 (PMC10104332; doi:10.1371/journal.pone.0283866)
Supplement: S14 Table — Values of ‘1’ indicate complete dominance of the row variable over the column variable. Values of ‘0.5’ indicate that complete dominance could not be established. (PDF) [file pone.0283866.s015.pdf]

S14 Table. Dominance Analysis Results Predicting the Antisocial Facet of Psychopathy.

		1	2	3	4	5	6	7	8	9	10
1	Positive Urgency	-	1	1	1	1	1	1	1	1	1
2	Delay Discounting		-	0.5	0.5	0.5	0.5	0.5	1	0.5	1
3	Decision Quality			-	0.5	0.5	0.5	0.5	0.5	0.5	1
4	False Alarms (GNG)				-	0.5	0.5	0.5	0.5	0.5	1
5	General Impulsivity					-	0.5	0.5	0.5	0.5	0.5
6	Sensation Seeking						-	0.5	0.5	0.5	0.5
7	Negative Urgency							-	0.5	0.5	0.5
8	IGT total								-	0.5	1
9	Lack of Premeditation									-	0.5
10	Commission Errors										-

Notes. Values of '1' indicates complete dominance of the row variable over the column variable.

Values of '0.5' indicate that complete dominance could not be established.
