# Supplementary material for: Identifying distinct profiles of impulsivity for the four facets of psychopathy
Source: PLoS One. 2023 Apr 14;18(4):e0283866. doi: 10.1371/journal.pone.0283866 (PMC10104332; doi:10.1371/journal.pone.0283866)
Supplement: S15 Table — Group indicates drug dependence such that 0 = non-dependent, 1 = dependent. (PDF) [file pone.0283866.s016.pdf]

S15 Table. Multiple Regression Model Including Group Interactions Predicting the Interpersonal Facet of Psychopathy.

Predictors	*Estimates*	*CI*	*p*
Negative Urgency	-0.15	-0.38 – 0.08	0.209
Positive Urgency	0.39	0.17 – 0.62	0.001
General Impulsivity	0.07	-0.16 – 0.31	0.535
Sensation Seeking	0.21	0.07 – 0.36	0.004
Lack of Premeditation	0.01	-0.16 – 0.18	0.911
Decision Quality	0.02	-0.13 – 0.16	0.837
Delay Discounting	0.22	0.09 – 0.34	0.001
Commission Errors	0.04	-0.08 – 0.16	0.515
Group	0.15	-0.03 – 0.34	0.095
Positive Urgency * Group	-0.06	-0.34 – 0.23	0.700
Negative Urgency * Group	0.04	-0.26 – 0.34	0.772
General Impulsivity * Group	-0.09	-0.40 – 0.22	0.578
Sensation Seeking * Group	-0.09	-0.29 – 0.10	0.332
Lack of Premeditation * Group	-0.03	-0.26 – 0.20	0.806
Decision Quality * Group	-0.16	-0.35 – 0.02	0.084
Delay Discounting * Group	-0.10	-0.27 – 0.06	0.226
Commission Errors * Group	-0.03	-0.20 – 0.14	0.721

Note. Group indicates drug dependence such that 0 = non-dependent, 1 = dependent).
