# Supplementary material for: Identifying distinct profiles of impulsivity for the four facets of psychopathy
Source: PLoS One. 2023 Apr 14;18(4):e0283866. doi: 10.1371/journal.pone.0283866 (PMC10104332; doi:10.1371/journal.pone.0283866)
Supplement: S16 Table — Group indicates drug dependence such that 0 = non-dependent, 1 = dependent. (PDF) [file pone.0283866.s017.pdf]

S16 Table. Multiple Regression Model Including Group Interactions Predicting the Affective Facet of Psychopathy.

Predictors	*Estimates*	*CI*	*p*
Negative Urgency	0.02	-0.20 – 0.25	0.847
Positive Urgency	0.29	0.08 – 0.51	0.008
General Impulsivity	0.02	-0.21 – 0.25	0.863
Sensation Seeking	0.14	-0.00 – 0.28	0.051
Lack of Premeditation	-0.01	-0.17 – 0.16	0.942
Decision Quality	-0.05	-0.19 – 0.09	0.474
Delay Discounting	0.21	0.09 – 0.33	0.001
Commission Errors	0.14	0.02 – 0.26	0.021
Group	0.19	0.01 – 0.36	0.037
Positive Urgency * Group	-0.18	-0.34 – -0.01	0.038
Negative Urgency * Group	0.18	-0.11 – 0.46	0.222
General Impulsivity * Group	-0.14	-0.43 – 0.15	0.356
Sensation Seeking * Group	0.01	-0.29 – 0.31	0.965
Lack of Premeditation * Group	-0.16	-0.35 – 0.03	0.094
Decision Quality * Group	0.02	-0.20 – 0.24	0.877
Delay Discounting * Group	-0.09	-0.27 – 0.09	0.331
Commission Errors * Group	-0.07	-0.23 – 0.10	0.420

Note. Group indicates drug dependence such that 0 = non-dependent, 1 = dependent).
