# Supplementary material for: Identifying distinct profiles of impulsivity for the four facets of psychopathy
Source: PLoS One. 2023 Apr 14;18(4):e0283866. doi: 10.1371/journal.pone.0283866 (PMC10104332; doi:10.1371/journal.pone.0283866)
Supplement: S17 Table — Group indicates drug dependence such that 0 = non-dependent, 1 = dependent. (PDF) [file pone.0283866.s018.pdf]

S17 Table. Multiple Regression Model Including Group Interactions Predicting the Lifestyle Facet of Psychopathy.

Predictors	*Estimates*	*CI*	*p*
Negative Urgency	0.09	-0.12 – 0.30	0.405
Positive Urgency	0.16	-0.04 – 0.37	0.124
General Impulsivity	0.30	0.09 – 0.52	0.006
Sensation Seeking	0.08	-0.05 – 0.21	0.247
Lack of Premeditation	0.11	-0.04 – 0.27	0.147
Decision Quality	0.05	-0.08 – 0.18	0.471
Delay Discounting	0.16	0.05 – 0.27	0.006
Commission Errors	0.05	-0.06 – 0.16	0.400
Group	0.11	-0.05 – 0.28	0.179
Positive Urgency * Group	-0.05	-0.21 – 0.10	0.504
Negative Urgency * Group	0.18	-0.08 – 0.45	0.171
General Impulsivity * Group	-0.04	-0.31 – 0.24	0.783
Sensation Seeking * Group	-0.07	-0.35 – 0.21	0.627
Lack of Premeditation * Group	-0.10	-0.28 – 0.08	0.261
Decision Quality * Group	-0.06	-0.27 – 0.14	0.546
Delay Discounting * Group	-0.16	-0.34 – 0.01	0.062
Commission Errors * Group	0.06	-0.10 – 0.21	0.469

Note. Group indicates drug dependence such that 0 = non-dependent, 1 = dependent).
