# Supplementary material for: Identifying distinct profiles of impulsivity for the four facets of psychopathy
Source: PLoS One. 2023 Apr 14;18(4):e0283866. doi: 10.1371/journal.pone.0283866 (PMC10104332; doi:10.1371/journal.pone.0283866)
Supplement: S18 Table — Group indicates drug dependence such that 0 = non-dependent, 1 = dependent. (PDF) [file pone.0283866.s019.pdf]

S18 Table. Multiple Regression Model Including Group Interactions Predicting the Antisocial Facet of Psychopathy.

Predictors	*Estimates*	*CI*	*p*
Negative Urgency	0.04	-0.18 – 0.26	0.693
Positive Urgency	0.38	0.17 – 0.59	<0.001
General Impulsivity	-0.06	-0.29 – 0.16	0.572
Sensation Seeking	0.19	0.05 – 0.33	0.008
Lack of Premeditation	0.12	-0.04 – 0.28	0.146
Decision Quality	-0.02	-0.16 – 0.11	0.732
Delay Discounting	0.16	0.04 – 0.27	0.009
Commission Errors	0.02	-0.10 – 0.13	0.746
Group	0.25	0.07 – 0.42	0.005
Positive Urgency * Group	-0.07	-0.23 – 0.09	0.408
Negative Urgency * Group	0.04	-0.23 – 0.32	0.753
General Impulsivity * Group	-0.08	-0.36 – 0.21	0.587
Sensation Seeking * Group	0.09	-0.20 – 0.38	0.551
Lack of Premeditation * Group	-0.17	-0.35 – 0.01	0.062
Decision Quality * Group	-0.13	-0.34 – 0.09	0.251
Delay Discounting * Group	-0.12	-0.30 – 0.05	0.172
Commission Errors * Group	0.01	-0.15 – 0.16	0.947

Note. Group indicates drug dependence such that 0 = non-dependent, 1 = dependent).
