# Supplementary material for: Identifying distinct profiles of impulsivity for the four facets of psychopathy
Source: PLoS One. 2023 Apr 14;18(4):e0283866. doi: 10.1371/journal.pone.0283866 (PMC10104332; doi:10.1371/journal.pone.0283866)
Supplement: S19 Table — Values of ‘1’ indicate complete dominance of the row variable over the column variable. Values of ‘0.5’ indicate that complete dominance could not be established. (PDF) [file pone.0283866.s020.pdf]

S19 Table. Multivariate Dominance Analysis Predicting All Four Facets of Psychopathy.

		1	2	3	4	5	6	7	8	P^2	R^2	Rank
1	Positive Urgency	-	0.5	1	1	1	1	1	1	.03	.12	1
2	General Impulsivity		-	0.5	1	1	1	1	1	.03	.11	2
3	Sensation Seeking			-	0.5	0.5	0.5	1	1	.01	.04	3
4	Commission Errors				-	0.5	0.5	0.5	1	.02	.02	4
5	Negative Urgency					-	0.5	0.5	0.5	.02	.08	5
6	Lack of Premeditation						-	0.5	0.5	.01	.04	5
7	Delay Discounting								0.5	.01	.02	5
8	Decision Quality								-	.01	.01	5

Notes. A value of 1 indicates complete dominance of the row variable over the column variable, 0.5 indicates that complete dominance could not be established (e.g., conditional dominance) and a value of 0 indicated complete dominance of the column variable over the row variable. R^2 and P^2 indicate the average contribution of each predictor across all subset models.
